# A brief screen for attention-deficit/hyperactivity disorder in the South African workplace

**DOI:** 10.4102/sajpsychiatry.v26i0.1500

**Published:** 2020-10-01

**Authors:** Charles van Wijk, Nazneen Firfirey

**Affiliations:** 1 Institute for Maritime Medicine, Simon’s Town, South Africa

## Background

Adult attention-deficit/hyperactivity disorder (ADHD) is a condition characterised by a persistent pattern of inattention and/or hyperactivity–impulsivity that demonstrably interferes with social, academic and/or occupational functioning.^1^ Established epidemiological reports for workplace samples suggest a prevalence of around 3.5% (1.2% – 7.3%, dependent on country) for ADHD amongst adults.^2,3^ Adult ADHD has been associated with significant functional impairment in the workplace and has been associated with a two-fold increased risk of accidents and workplace injuries.^2,4,5^

Adult ADHD remains underdiagnosed, excluding individuals from mental healthcare that may support their safe employment.^6^ One reason for its under-recognition is the high prevalence of comorbid conditions in adults with ADHD, particularly mood, anxiety and substance use disorders.^1,7,8,9^ The symptoms associated with such disorders are often the primary complaint, and unless specifically asked, problems with attention and focus are unlikely to be reported spontaneously.^10^ Further, adults with ADHD may develop compensatory mechanisms for the workplace and less often report their symptoms as affecting their occupational functioning.^11^

During diagnostic interviews, clinicians typically screen for disorders that are comorbid with the principal diagnosis by asking about the comorbid disorder’s necessary feature or ‘gate-criterion’. For example, in a patient with a principal diagnosis of major depressive disorder, a clinician might inquire about the presence of panic attacks or excessive worry. In contrast, for polythetically defined disorders such as ADHD, there is no ‘gate-criterion’ because diagnosis is based on the presence of a minimum number of attributes from a longer list and no single attribute is required to be present.^10^ Thus, a barrier to recognise ADHD is the large number of criteria that must be considered to rule in or rule out the disorder.^10^

Given the under-recognition of ADHD, as well as the risk of workplace accidents and injuries, screening as part of occupational mental health surveillance may be beneficial. Although ADHD is always a clinical diagnosis, informed by a comprehensive assessment, multiple scales are available for screening.^12,13,14^ However, questionnaires may be lengthy (thus demanding sustained attention, which inherently puts individuals who struggle to remain focused, at a disadvantage), based on outdated diagnostic criteria or not validated for workers’ home language and thus impractical in workplace mental health surveillance contexts. Given the scarcity of resources for mental health services, amongst the other limited resources for large scale screening of workers, there is a need to consider brief screening tools to identify individuals who may benefit from referral for further assessment.

Previous analysis of data from the Rhode Island Methods to Improve Diagnostic Assessment and Services (RIMDAS) project in the USA (using psychiatric outpatients) indicated the usefulness of two features of ADHD to serve as ‘gate-criteria’ to screen for the presence of ADHD in adults. It identified a 2-item screen, namely difficulty sustaining attention (Diagnostic and Statistical Manual of Mental Disorders [DSM–5] criterion A1b) and fidgetiness (DSM-5 criterion A2a), with a reported sensitivity of > 0.90 and negative predictive value (NPV) of > 0.97, to screen for adult ADHD.^10^

### Aim

This study aimed to replicate the analysis of Zimmerman et al.,^10^ using data from local South African (SA) workplace samples. This was to determine whether it would be possible to similarly identify one or two ADHD symptoms that could serve as ‘gate-criteria’ to screen for ADHD in workplace populations, to improve recognition within the SA occupational medicine context.

## Method

### Study design and setting

The dataset came from a previously reported survey of adult ADHD in SA workplace populations.^3^ Data were collected through occupational mental health surveillance initiatives.

### Sample

**FIGURE 1:**
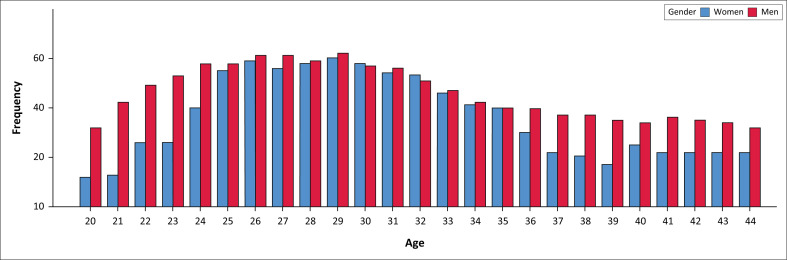
Gender and age distribution of sample.

The current sample consisted of 2048 workers (43.9% women) in full-time employment, with ages between 19 and 45 (*M* = 31.0 ± 6.4). As a result of the diagnostic requirement of onset of symptoms in childhood, the assessment was limited to respondents under the age of 45 because of concerns about the accuracy of retrospective recall amongst older respondents.^7^ Age by gender distribution is presented in Figure 1. Participants were drawn from across different sites and occupational contexts and considered ‘skilled labour’, with all reporting some form of vocational training. Detailed sample composition has been reported previously.^3^

### Measurement

All participants were interviewed by clinical psychologists, using a DSM-5 based schedule of 21 items, enquiring into the nine symptoms of inattention and the nine symptoms of hyperactivity and impulsivity (criterion A), time of onset (criterion B), presence in multiple settings (criterion C) and evidence of impact on functioning (criterion D). Collateral information was not sourced and outcomes were indicative of diagnostic likelihood, rather than final diagnosis. Data on comorbidities were not available for this analysis.

### Data analysis

For the present study, the 18 items reflecting DSM-5 symptoms were subjected to a receiver operating characteristics (ROC) curve analysis, using SPSS-25. Further, sensitivity and specificity, as well as positive predictive value (PPV) and NPV, were computed for each item.

Following the study by Zimmerman et al., the primary interest was in each symptom’s sensitivity (i.e. likelihood that a patient with ADHD has the criterion) and NPV (i.e. likelihood that a patient who does not have the criterion is not diagnosed with ADHD). A symptom or diagnostic criterion functions well as a screen if almost all patients with the disorder have the symptom (i.e. high sensitivity) and most patients who screen negative do not have the disorder (i.e. high NPV). Thus, for screening purposes, having high PPV (i.e. high likelihood of having the disorder if the criterion is present) is less critical because the quick screen is anticipated to be followed by a comprehensive diagnostic assessment.^10^

### Ethical consideration

The data were collected after approval from Stellenbosch University Health Research Ethics Committee (#N18/03/039). Workers were invited to participate on a voluntary basis, provided informed consent and all data were anonymised prior to inclusion in this analysis.

## Results

Frequency of likely DSM-5 adult ADHD was 3.2% (66 of the sample). Area under the curve (AUC), as well as sensitivity, specificity, PPV and NPV for the 18 symptoms are presented in Table 1. All 18 symptoms showed acceptable AUC and NPV, with varying sensitivities and specificities.

**TABLE 1:** Item characteristics.

DSM-5 criterion A	AUC	Sensitivity	Specificity	PPV	NPV
1 a	0.88	0.83	0.86	0.12	1.00
1 b	0.90	0.93	0.76	0.08	1.00
1 c	0.88	0.79	0.83	0.11	0.99
1 d	0.88	0.71	0.73	0.18	0.10
1 e	0.84	0.64	0.89	0.12	0.99
1 f	0.82	0.69	0.84	0.08	0.99
1 g	0.87	0.83	0.81	0.09	1.00
1 h	0.90	0.89	0.74	0.07	1.00
1 i	0.85	0.60	0.88	0.11	0.99
2 a	0.89	0.93	0.78	0.08	1.00
2 b	0.87	0.62	0.92	0.15	0.99
2 c	0.86	0.83	0.60	0.05	1.00
2 d	0.77	0.57	0.82	0.06	0.99
2 e	0.89	0.88	0.80	0.07	1.00
2 f	0.84	0.85	0.73	0.06	1.00
2 g	0.82	0.76	0.76	0.06	0.99
2 h	0.86	0.81	0.74	0.07	1.00
2 i	0.82	0.69	0.86	0.10	0.99

AUC, area under the curve; PPV, positive predictive value; NPV, negative predictive value.

Four symptoms were identified that met the requirements of high AUC, high sensitivity and high NPV (i.e.Criterion A 1b and 1h, and 2a and 2e). Of those, the two with the highest sensitivity were similar to the features reported from the RIMDAS sample, namely difficulty in sustaining attention and fidgetiness. Both had a sensitivity > 0.90 and a NPV > 0.95.

## Discussion

The frequency of adult ADHD in this sample is consistent with the reports from other workplace samples.^2^ This study also closely replicated previous analyses of North American data from a psychiatric cohort, with similar test-characteristics found for the two pertinent items. The 2-item screen identified from the South African data – difficulty in sustaining attention and fidgetiness – had a sensitivity of > 90% and NPV of > 95%. In this dataset from SA workplace populations, the presence of these two features appeared to capture most individuals with a likely diagnosis of adult ADHD, whereas its absence ruled out the disorder. The identification of these two symptoms are consistent with other studies, which described difficulty in sustaining attention as amongst the most frequent ADHD criteria and fidgetiness as the most frequent hyperactivity-impulsivity symptom.^15,16,17^

The two features thus appear to have potential as ‘gate-criteria’ to screen for adult ADHD as part of occupational mental health surveillance. This may enable up-scaling the screening of worker populations to include ADHD, to facilitate the referral of identified individuals for further assessment and management. It would be important to position such screening within the occupational medicine context,^18^ to mitigate the risk that such screening may be perceived as punitive, rather than therapeutically supportive. Workers may be reluctant to disclose symptoms if they perceive it to threaten their continued employment, whilst they may be reassured of its positive medical benefit if perceived as part of ongoing occupational health support and personal safety management.

Furthermore, it is important to distinguish between screening and case identification. These findings do not suggest that the diagnosis of ADHD can be abbreviated to an assessment of the presence or absence of difficulty sustaining attention and fidgetiness.^10^ The PPV of the two items were very low, and the majority of workers who reported either of these symptoms did not receive a diagnosis of ADHD. In contrast to the low PPV, the NPV was > 95%, and in workplace contexts similar to the study setting, clinicians may be confident in ruling out a diagnosis of ADHD in patients who do not report either of these two criteria.

It is emphasised that adult ADHD is always a clinical diagnosis informed by a comprehensive assessment. Brief screens like these are never meant to replace longer scales or clinical examination and in the occupational health context serve only to screen workers in order to stream them to appropriate mental health services. Accurate identification facilitates appropriate referral for therapeutic management, for which established local guidelines are available.^14^

### Limitations

The data represented generally healthy and skilled worker samples and cannot necessarily be generalised to community samples, psychiatric outpatients or unskilled labour populations without further research. Further, diagnostic likelihood depended on self-report, and future studies may need to include collateral information to confirm diagnosis. As a result of a technical error, data on comorbidities were not available for this analysis, and will need to be included in future studies. Difficulties with sustaining attention or remaining still could also occur as expressions of other conditions, which again emphasises the need for a comprehensive assessment to establish a diagnosis of ADHD.

## Conclusion

Clinicians, in comparable workplace contexts, can now with some confidence use two questions to screen for the possible presence of adult ADHD, when conducting diagnostic assessments. This can be performed with a 2-item screen that probes difficulty in sustaining attention and fidgetiness, where at least one is present in most patients with the disorder and the absence of which effectively rules out the disorder. The psychometric properties of the 2-item screen for ADHD suggest that it can function as such a screen. Such screening would then allow for streaming to appropriate mental health support.
